# A Ferroptosis and Pyroptosis Molecular Subtype-Related Signature Applicable for Prognosis and Immune Microenvironment Estimation in Hepatocellular Carcinoma

**DOI:** 10.3389/fcell.2021.761839

**Published:** 2021-11-15

**Authors:** Junyu Huo, Jinzhen Cai, Ge Guan, Huan Liu, Liqun Wu

**Affiliations:** Liver Disease Center, The Affiliated Hospital of Qingdao University, Qingdao, China

**Keywords:** hepatocellular carcinoma, ferroptosis, pyroptosis, prognostic, signature

## Abstract

**Background:** Due to the heterogeneity of tumors and the complexity of the immune microenvironment, the specific role of ferroptosis and pyroptosis in hepatocellular carcinoma (HCC) is not fully understood, especially its impact on prognosis.

**Methods:** The training set (*n* = 609, merged by TCGA and GSE14520) was clustered into three subtypes (C1, C2, and C3) based on the prognosis-related genes associated with ferroptosis and pyroptosis. The intersecting differentially expressed genes (DEGs) among C1, C2, and C3 were used in univariate Cox and LASSO penalized Cox regression analysis for the construction of the risk score. The median risk score served as the unified cutoff to divide patients into high- and low-risk groups.

**Results:** Internal (TCGA, *n* = 370; GSE14520, *n* = 239) and external validation (ICGC, *n* = 231) suggested that the 12-gene risk score had high accuracy in predicting the OS, DSS, DFS, PFS, and RFS of HCC. As an independent prognostic indicator, the risk score could be applicable for patients with different clinical features tested by subgroup (*n* = 26) survival analysis. In the high-risk patients with a lower infiltration abundance of activated B cells, activated CD8 T cells, eosinophils, and type I T helper cells and a higher infiltration abundance of immature dendritic cells, the cytolytic activity, HLA, inflammation promotion, and type I IFN response in the high-risk group were weaker. The TP53 mutation rate, TMB, and CSC characteristics in the high-risk group were significantly higher than those in the low-risk group. Low-risk patients have active metabolic activity and a more robust immune response. The high- and low-risk groups differed significantly in histology grade, vascular tumor cell type, AFP, new tumor event after initial treatment, main tumor size, cirrhosis, TNM stage, BCLC stage, and CLIP score.

**Conclusion:** The ferroptosis and pyroptosis molecular subtype-related signature identified and validated in this work is applicable for prognosis prediction, immune microenvironment estimation, stem cell characteristics, and clinical feature assessment in HCC.

## Background

Programmed cell death (PCD) refers to an active death process that occurs to maintain the stability of the internal environment after a cell receives a certain signal or is stimulated by some factors; it occurs not only in the normal development of individuals but also in abnormal physiological states or diseases (Moujalled et al., 2021). Among the types of PCD, ferroptosis, and pyroptosis, two new PCD modes confirmed in recent years, are significantly different from other types of death modes, such as apoptosis, necrosis, and autophagy in their cell death pathway and related morphological and biochemical characteristics, which has attracted increasing amounts of attention (Wiernicki et al., 2020).

The occurrence of pyroptosis mainly depends on inflammatory caspases and the GSDM protein family (Bergsbaken et al., 2009). In short, activated caspase cleaves GSDM proteins and releases their N-terminal domain, which binds to membrane lipids and makes holes in the cell membrane. This causes a change in cell osmotic pressure, and the cell swells until its membrane ruptures, which leads to the release of the cell contents. The released contents activate a strong inflammatory response, leading to programmed cell necrosis of neighboring cells (Liu et al., 2021). The pathological features of ferroptosis are mainly the accumulation of iron-dependent lipid peroxides, and the typical morphological manifestations are cell volume shrinkage and an increase in mitochondrial membrane area. The mechanism is mainly related to a disorder of iron metabolism, an imbalance of the amino acid antioxidant system and the accumulation of lipid peroxide (Dixon et al., 2012).

Hepatocellular carcinoma (HCC) is the most common and aggressive primary tumor in the liver, with limited treatment options (El-Serag, 2007). Surgical resection is one of the standard treatments; however, HCC still shows a high postoperative recurrence rate, rapid progression, and a poor prognosis (Villanueva et al., 2007), and there is an urgent need to find new therapeutic targets for HCC. Ferroptosis and pyroptosis play important roles in the occurrence and development of HCC. In the precancerous stage, normal hepatocyte pyroptosis promotes the process of liver fibrosis (Gaul et al., 2021). The accumulation of inflammasomes and inflammatory factors will aggravate the transformation from liver cirrhosis to primary liver cancer (Wree et al., 2014). After the formation of cancer cells in the tumor stage, inflammasomes and pyroptosis are inhibited, forming an internal malignant tumor microenvironment, blocking the death of cancer cells and accelerating the development of primary liver cancer to malignancy (Chu et al., 2016; Wei et al., 2019). Sorafenib is a multikinase inhibitor that improves the survival rate of patients with advanced HCC to a certain extent (Llovet et al., 2008). The latest experimental study found that its antitumor effect not only blocks the proliferation and antiangiogenesis of cancer cells but also is related to inducing ferroptosis (Louandre et al., 2015; Sun et al., 2016).

The involvement of ferroptosis and pyroptosis provides a new research direction for the treatment of HCC, but the relevant research is still insufficient, especially in terms of its impact on the clinical outcome of HCC. In addition, whether there are other molecular pathways in the occurrence and development of ferroptosis and pyroptosis remains to be studied. Therefore, further exploration of the mechanism and the related signal transduction pathways of ferroptosis and pyroptosis in HCC will help clarify the pathological mechanism of HCC and find new drug targets to provide new ideas and strategies for the prevention and treatment of HCC.

## Materials and Methods

### Data Acquisition

The mRNA expression data and clinical information of three independent HCC cohorts (TCGA-LIHC, *n* = 370; GSE14520, *n* = 239; ICGC-LIRI-JP, *n* = 231) were downloaded from three public databases: The Cancer Genome Atlas (TCGA),^1^ the International Cancer Genomics Consortium (ICGC),^2^ and the Gene Expression Omnibus (GEO).^3^ The usage guidelines of the TCGA, ICGC, and GEO databases were fully satisfied for data collection. For data normalization, the values of the fragments per kilobase of transcript per million (FPKM) data (FPKM) in the three RNA-seq cohorts were transformed into transcripts per million kilobase (TPM) values using the R package “limma,” and the batch effect in different datasets was removed using the ComBat function of the R “SVA” package (Huo et al., 2021c,d). Because this study was based on a public database, approval of the local ethics committee was not required. The work flow of our work is shown in Supplementary Material 1.

### Ferroptosis- and Pyroptosis-Related Gene Cluster Analysis

Ferroptosis- and pyroptosis-related genes (*n* = 313, Supplementary Material 2) were obtained from the molecular signature database (MSigDB^4^), FerrDb^5^ (Zhou and Bao, 2020), and previously published articles (Ye et al., 2021). TCGA and GSE14520 were merged into one training cohort (*n* = 609) to analyze the prognostic value of the related genes for HCC. Genes with a *p*-value < 0.05 tested by univariate Cox regression analysis were considered prognostic-related genes (PRGs). Then, the PRGs were subjected to cluster analysis by the R package “ConsensusClusterPlus.” The Kaplan–Meier method was used to analyze the overall survival (OS) difference between different subtypes. The differentially expressed genes (DEGs) between different subtypes were identified by the R package “limma” (fdr < 0.001) (Huo et al., 2021b). The intersecting DEGs were extracted for subsequent analysis.

### Building a Risk Score for Predicting the Overall Survival of Hepatocellular Carcinoma in the Training Cohort

Univariate Cox regression analysis was used to identify genes associated with OS in the training cohort (*n* = 609), and the filter *p*-value was set as < 0.05. The overfitting between the prognosis-related genes was removed by the least absolute shrinkage and selection operator (LASSO) algorithm to reduce the scope of the prognosis-related genes with penalty parameter tuning performed *via* 10−fold cross−validation based on the R package “glmnet” (Huo et al., 2021a). The genes with non-zero regression coefficients obtained from LASSO regression analysis were included in the multivariate Cox regression analysis (Huo et al., 2021g). The risk score was established by the expression level of each gene multiplied by its corresponding regression coefficients derived from multivariate Cox regression analysis of each gene (Huo et al., 2021e). The patients were divided into high- and low-risk groups according to the median risk score (Huo et al., 2021f). The Kaplan–Meier survival curves and the time-dependent receiver operational feature curves (ROC) generated with the R packages “suvminer” and “survival ROC” were used to assess the performance of the risk score in predicting the survival of patients with HCC.

### Evaluation of the Tumor Immune Microenvironment Using the Single Sample Gene Set Enrichment Analysis Algorithm

The single sample gene set enrichment analysis (ssGSEA) algorithm was used to quantify the infiltration abundance of 23 types of immune cells and the activity of 13 types of immune function pathways by the normalized enrichment score (NES) (Barbie et al., 2009). The differences in NES between the high- and low-risk groups were compared by independent-samples *t*-tests, and *p* < 0.05 was defined as statistically significant.

### Exploration of the Molecular Mechanism Underlying the Prognostic Signature

The activity of molecular pathways was quantified by the NES calculated from the gene set variation analysis (GSVA) method based on the “GSVA” R package (Hnzelmann et al., 2013). The differences in NES between the high- and low-risk groups were compared by independent-samples *t*-tests, and *p* < 0.05 was defined as statistically significant. The DEGs between the high- and low-risk groups were identified by the “limma” R package (fdr < 0.05), and the R package “clusterProfiler” was used for the annotation of the Gene Ontology (GO) function of DEGs. Gene set enrichment analysis (GSEA) was used to estimate the immune response between different risk groups, and *p*-value < 0.05 and FDR < 0.25 were considered to be different (Subramanian et al., 2005; Huo et al., 2021c).

### Exploration of the Genomics Landscape and Stemness Characteristics of the Prognostic Signature

The somatic mutation data, which were downloaded from TCGA GDC Data Portal (see text footnote 1), were used to compare the gene mutation rate and tumor mutation burden (TMB) in different risk groups. The mRNAsi is a quantitative index reflecting the stem cell characteristics of cancer cells calculated based on gene expression data; the closer its value is to 1, the lower the differentiation degree of cancer cells and the stronger the stem cell characteristics (Malta et al., 2018). The impact of gene mutation, TMB, and mRNAsi on prognosis was evaluated by Kaplan–Meier survival analysis. The mRNAsi and TMB differences between the high- and low-risk groups were compared by the independent-samples *t*-test, and the Spearman correlation test was used to analyze the correlation between the risk score and mRNAsi and TMB; *p* < 0.05 was suggested to indicate statistical significance.

### Internal and External Validation of the Prognostic Signature and Clinical Correlation Analysis

Internal validation included validation in the respective cohorts of TCGA (*n* = 370) and GSE14520 (*n* = 239) and subgroup (*n* = 26) validation according to clinical characteristics. External validation was conducted in the ICGC cohort (*n* = 231). The prognostic prediction performance of the risk score was assessed by Kaplan–Meier survival curves and time-dependent receiver operational feature (ROC) curves. Whether the risk score could be regarded as an independent prognostic indicator was determined by univariate and multivariate Cox regression analysis. The clinical features in different risk groups were compared by the chi square test. We calculated the IC50 of anticancer drugs by using R package pRRophetic and compared the difference of anticancer drug sensitivity between the high-risk group and the low-risk group.

## Results

### Ferroptosis and Pyroptosis Molecular Subtype Was Related to the Prognosis of Hepatocellular Carcinoma

**FIGURE 1:**
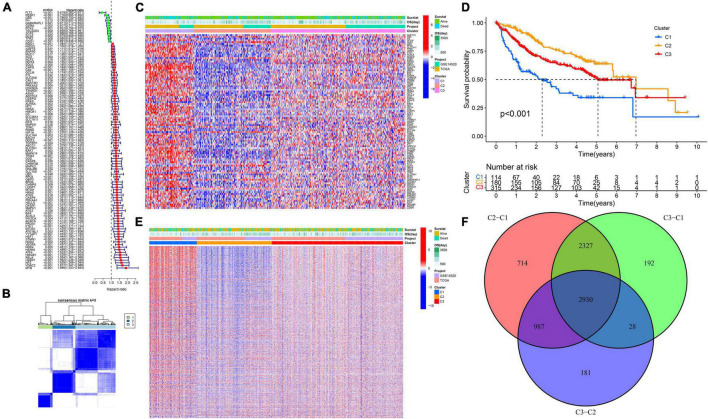
Ferroptosis- and pyroptosis-related genes cluster analysis. **(A)** The forest plot of PRGs. **(B,C)** Cluster analysis. **(D)** The Kaplan–Meier survival analysis. **(E)** The heatmap of DEGs. **(F)** The Venn plot of intersection DEGs.

A total of 99 genes were suggested to be associated with the OS of HCC by univariate Cox regression analysis (Figure 1A). We found that the intergroup correlations were lowest and the intragroup correlations were the highest when *k* = 3 by increasing the clustering variable (*k*) from 2 to 9, indicating that the training cohort (*n* = 609, merged by TCGA and GSE14520) could be well clustered into three subtypes based on the 99 prognostic related genes associated with ferroptosis and pyroptosis (Figures 1B,C). There were significant differences in OS among the three subtypes (Figure 1D). Next, we identified DEGs between different subtypes (Figure 1E), and a total of 2,930 intersecting DEGs were extracted for subsequent analysis (Figure 1F).

### A 12-Gene Prognostic Signature Established in the Training Cohort

**FIGURE 2:**
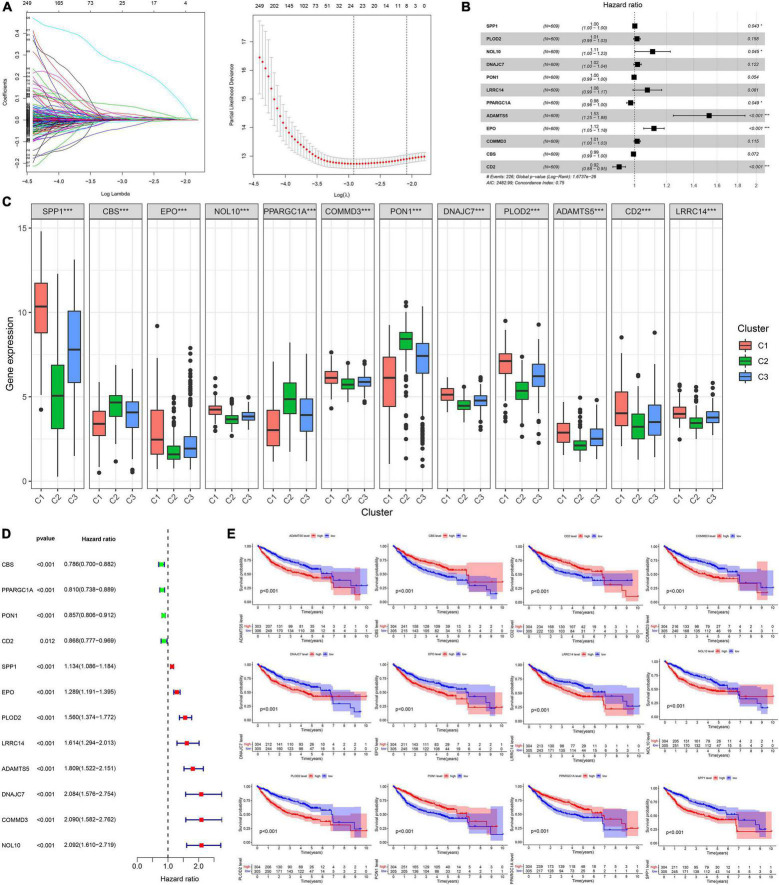
Building process of the prognostic signature in the training cohort. **(A,B)** LASSO and multivariate Cox regression analysis; *p < 0.05. **(C)** The boxplot of 12 genes in different subtypes; ***p < 0.001. **(D)** The forest plot of univariate Cox analysis. **(E)** The Kaplan–Meier survival analysis.

**FIGURE 3:**
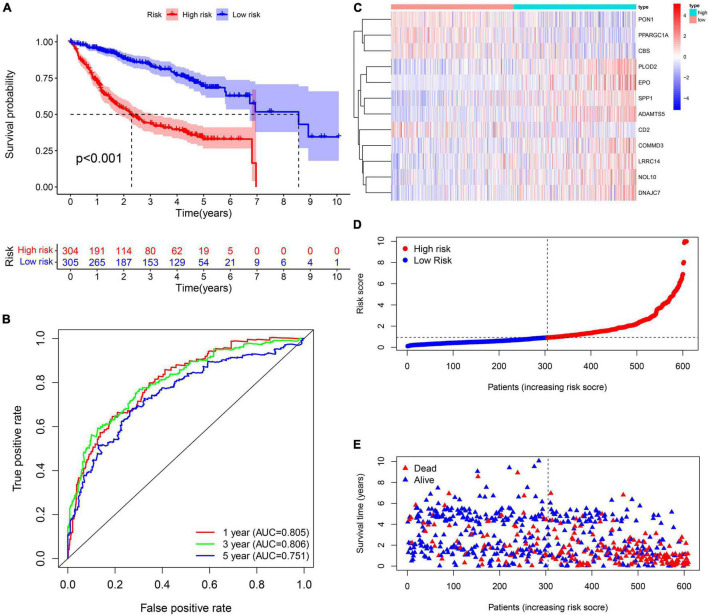
Prognostic assessment of the prognostic signature in the training cohort. **(A,B)** The Kaplan–Meier survival analysis and the time-dependent ROC analysis for the risk score in predicting the OS of patients in the training cohort. **(C–E)** The heatmap, risk score distribution, and survival status of patients in the training cohort.

A total of 2,376 intersecting DEGs were suggested to be associated with prognosis by univariate Cox regression analysis (*p* < 0.05) (Supplementary Material 3). Genes with zero LASSO regression coefficients were excluded (Figure 2A), and a 12-gene risk score was built by multivariate Cox regression analysis: ADAMTS5 × 0.424958696—CBS × 0.00624125 −CD2^∗^0.087852292 + COMMD3 × 0.014626568 + DNAJC7 × 0.016821996 + EPO × 0.110519557 + LRRC14 × 0.072966715 + NOL10 × 0.102824383 + PLOD2 × 0.013695569 − PON1 ×0.002910868 − —PPARGC1A × 0.022370895 + SPP1 × 0.001368761 (Figure 2B). The 12 genes were expressed significantly differentially among the three subtypes (Figure 2C). Univariate Cox regression analysis and Kaplan–Meier survival analysis showed that 4 of the 12 genes were prognostic protective factors and 8 genes were prognostic risk factors (Figures 2D,E). The OS of the high-risk patients with a risk score > 0.9368 was significantly reduced compared to that of the low-risk patients with a risk score < 0.9368 (*p* < 0.001, Figure 3A). The AUC (area under the curve) values of 1-, 3-, and 5-year OS predicted by the risk score were 0.805, 0.806, and 0.751, respectively (Figure 3B). The number of surviving patients decreased with an increasing risk score (Figures 3C–E).

### Internal Validation of the Prognostic Signature

**FIGURE 4:**
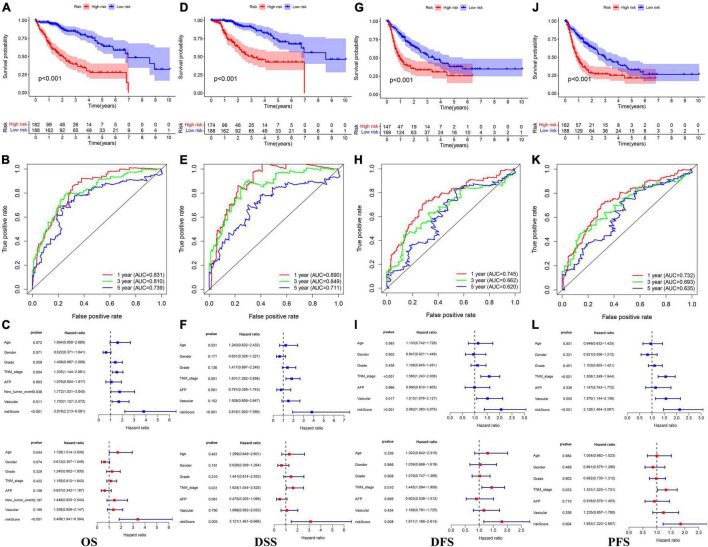
The Kaplan–Meier survival analysis, the time-dependent ROC analysis, and the Cox analysis of the risk score in the TCGA cohort **(A–C)** OS, **(D–F)** DSS, **(G–I)** DFS, and **(J–L)** PFS. *For **(C,F,I,L)** blue represents univariate Cox analysis, red represent multivariate Cox analysis.

**FIGURE 5:**
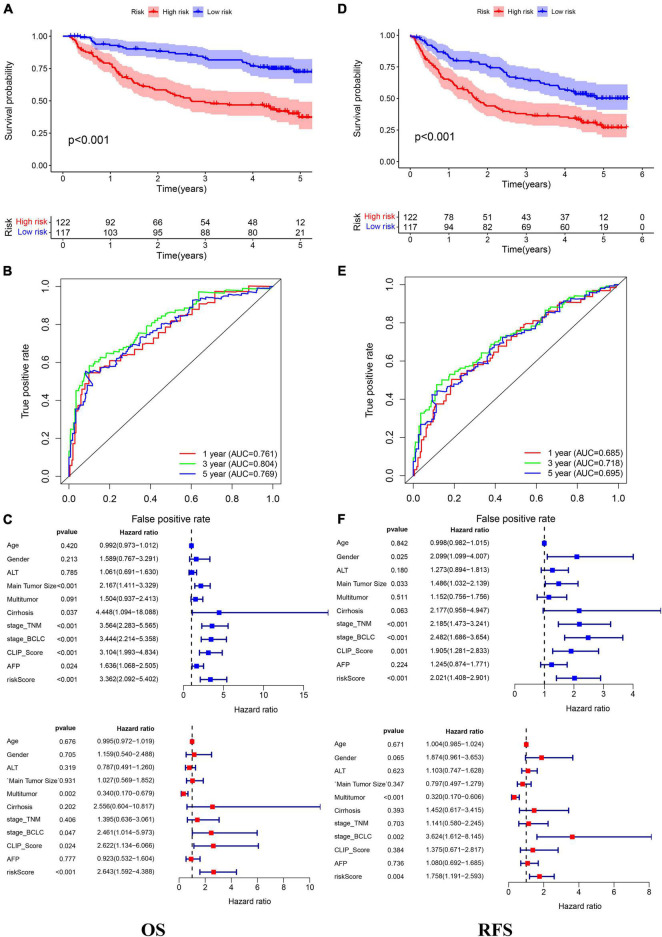
The Kaplan–Meier survival analysis, the time-dependent ROC analysis, and the Cox analysis of the risk score in the GSE14520 cohort **(A–C)** OS and **(D–F)** RFS. *For **(C,F)** blue represent univariate Cox analysis, red represent multivariate Cox analysis.

**FIGURE 6:**
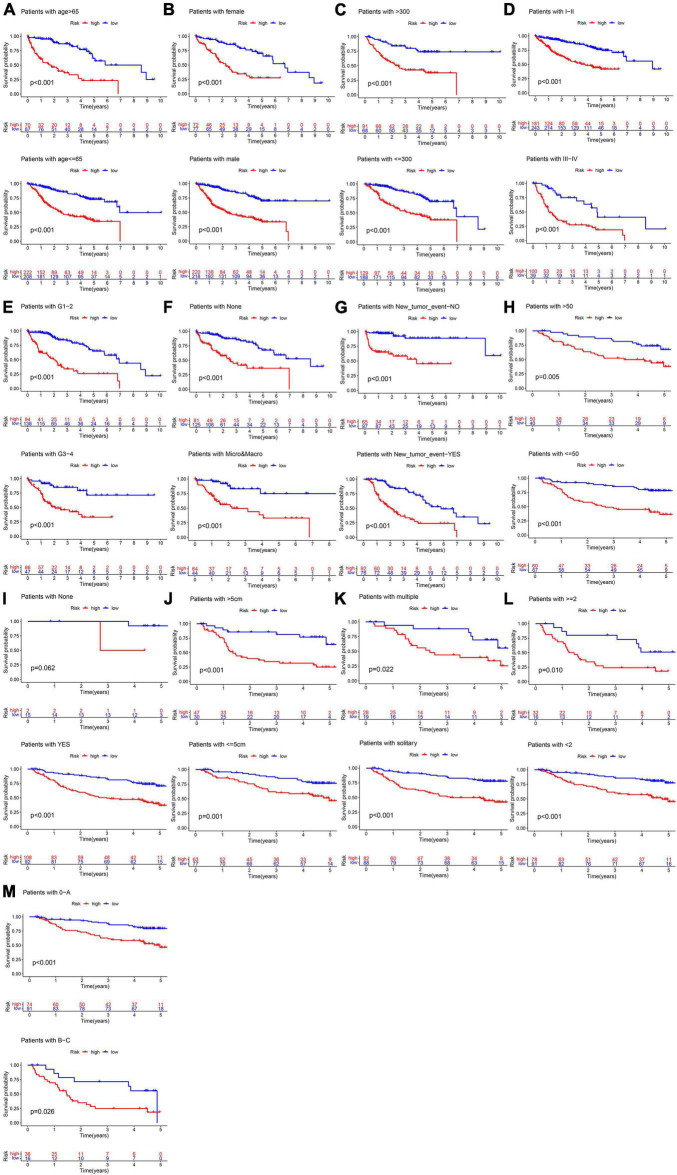
Subgroup survival analysis in the training cohort. **(A)** Age. **(B)** Gender. **(C)** AFP (ng/ml). **(D)** TNM stage. **(E)** Histology grade. **(F)** Vascular tumor cell type. **(G)** New tumor event after initiative treatment. **(H)** ALT. **(I)** Cirrhosis. **(J)** Main tumor size. **(K)** Tumor type. **(L)** CLIP_Score. **(M)** BCLC stage.

In the TCGA cohort, the OS, disease-specific survival (DSS), disease-free survival (DFS), and progression-free survival (PFS) in the high-risk group were all significantly lower than those in the low-risk group (Figures 4A,D,G,J). The AUC values of 1-, 3-, and 5-year OS predicted by the risk score were 0.831, 0.810, and 0.739, respectively (Figure 4B). The AUC values of 1-, 3-, and 5-year DSS predicted by the risk score were 0.890, 0.849, and 0.711, respectively (Figure 4E). The AUC values of 1-, 3-, and 5-year DFS predicted by the risk score were 0.745, 0.662, and 0.620, respectively (Figure 4H). The AUC values of 1-, 3-, and 5-year PFS predicted by the risk score were 0.732, 0.693, and 0.635, respectively (Figure 4K). Univariate and multivariate Cox regression analyses suggested that the risk score was an independent predictor for OS, DSS, DFS, and PFS (Figures 4C,F,I,L). In the GSE14520 cohort, the OS and relapse-free survival (RFS) in the high-risk group were all significantly reduced compared to those in the low-risk group (Figures 5A,D). The AUC values of 1-, 3-, and 5-year OS predicted by the risk score were 0.761, 0.804, and 0.769, respectively (Figure 5B). The AUC values of 1-, 3-, and 5-year RFS predicted by the risk score were 0.685, 0.718, and 0.695, respectively (Figure 5E). The risk score was an independent predictor of OS and RFS, as suggested by univariate and multivariate Cox regression analysis (Figures 5C,F). In each subgroup (*n* = 26) assigned by clinical characteristics, the OS of the high-risk group was significantly reduced (Figures 6A–M).

### Comparison of the Immune Microenvironment in Different Risk Groups

**FIGURE 7:**
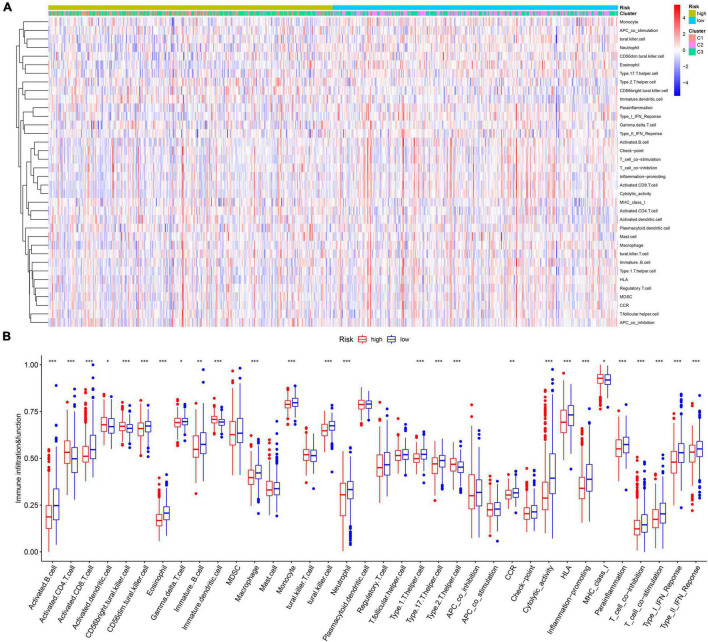
The landscape of tumor immune microenvironment in the training cohort. **(A)** The heatmap. **(B)** The boxplot of the TIME difference between high- and low-risk groups. *p < 0.05, **p < 0.01, ***p < 0.001.

**FIGURE 8:**
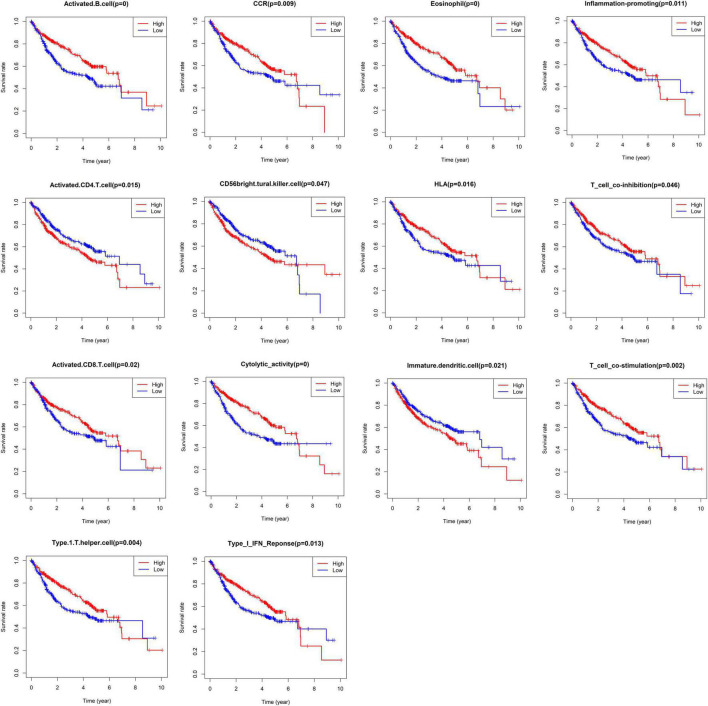
The Kaplan–Meier survival analysis regarding immune cells infiltration and immune function.

The infiltration abundances of activated B cells, activated CD8 T cells, eosinophils, and type I T helper cells in the low-risk group were significantly higher than those in the high-risk group (Figures 7A,B), and the infiltration abundances of activated CD4 T cells, CD56 natural killer cells, and immature dendritic cells (DCs) in the high-risk group were significantly higher than those in the low-risk group (Figures 7A,B). The activity of CCR, cytolytic activity, HLA, inflammation promotion, T-cell coinhibition, T-cell costimulation, and type I IFN response in the low-risk group were significantly higher than those in the high-risk group (Figures 4C,D). The Kaplan–Meier survival analysis showed that the patients with a higher infiltration abundance of activated CD4 T cells, CD56 natural killer cells, and immature DCs had a poor prognosis (*p* < 0.05, Figure 8), while patients with a higher infiltration abundance of activated B cells, activated CD8 T cells, eosinophils, and type I T helper cells had a better prognosis (*p* < 0.05, Figure 8). The patients with stronger activity of CCR, cytolytic activity, HLA, inflammation promoting, T cell coinhibition, T cell costimulation, and Type I IFN response had a better prognosis (*p* < 0.05, Figure 8).

### Correlation Analysis Between Risk Score and Gene Mutation, Tumor Mutation Burden, and Stemness Indices (mRNAsi)

**FIGURE 9:**
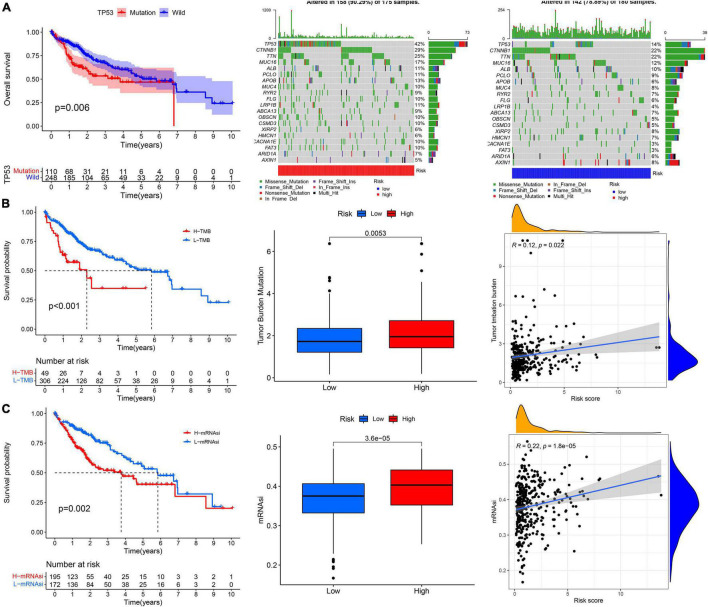
The correlation analysis between the risk score and **(A)** TP53 mutation, **(B)** tumor mutation burden, and **(C)** cancer stem cells indices (mRNAsi).

The prognosis of patients with TP53 mutations, higher TMB, and higher stemness indices (mRNAsi) was significantly worse (Figures 9A–C). The high-risk group had a higher TP53 mutation rate than the low-risk group (42 vs. 14%, Figures 9A–C). The TMB and mRNAsi significantly increased in the high-risk group (Figures 9A–C), and the risk score showed a positive correlation with the TMB and mRNAsi (Figures 9A–C).

### The Potential Molecular Mechanism of the Prognostic Signature

**FIGURE 10:**
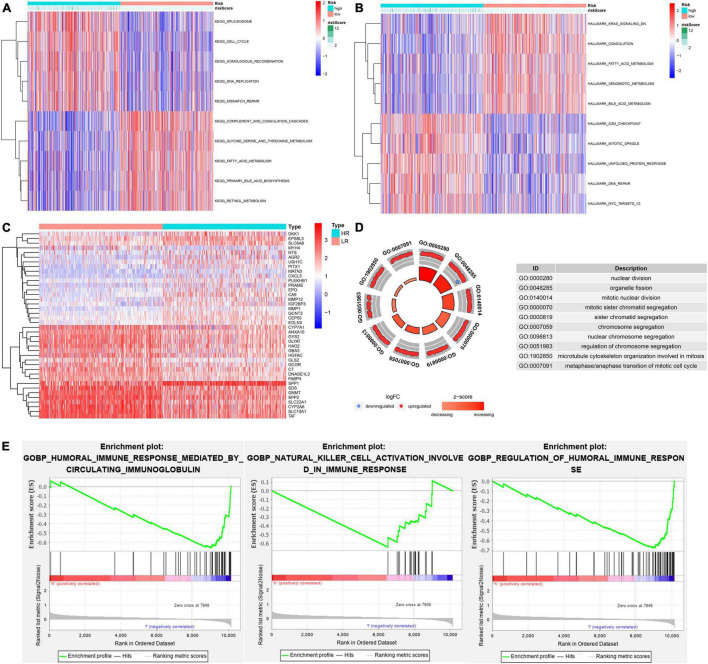
The potential molecular mechanism of the prognostic signature. **(A)** GSVA-KEGG. **(B)** GSVA-HALLMARK. **(C)** The heatmap of DEGs between high- and low-risk groups. **(D)** GO function annotation of DEGs. **(E)** GSEA using immune gene set.

**FIGURE 11:**
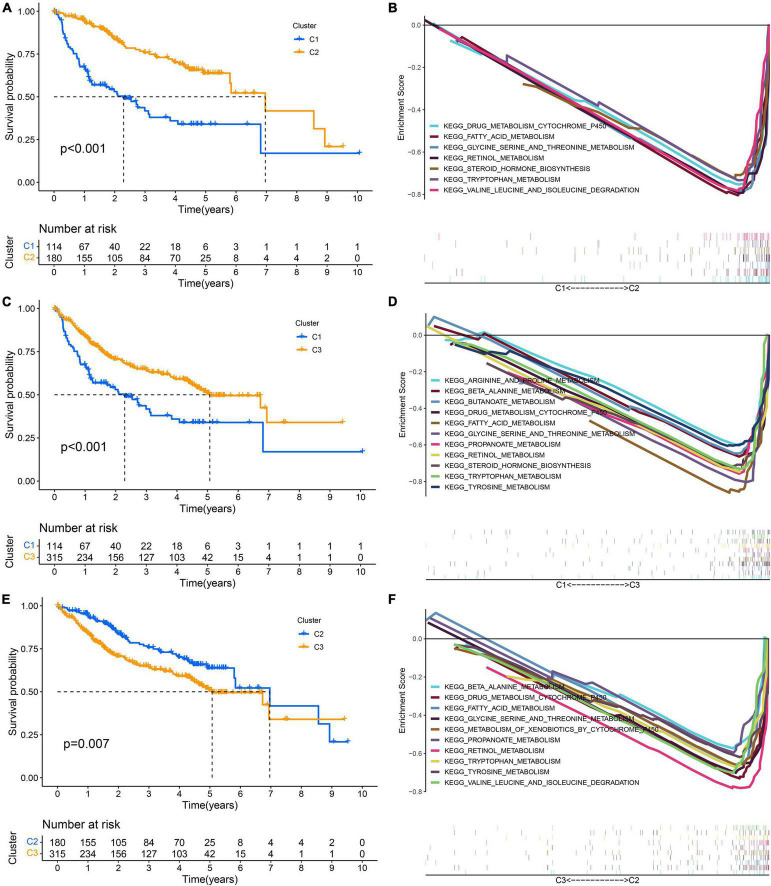
Pairwise comparison of metabolism activity among the three subtypes. **(A,B)** C1–C2. **(C,D)** C1–C3. **(E,F)** C2–C3.

GSVA showed that pathways related to the cell cycle, such as the G2/M checkpoint and mitotic spindle, and those associated with DNA repair, such as homologous recombination and mismatch repair, were significantly active in the high-risk group, while the activity of pathways related to metabolism, such as fatty acid metabolism, bile acid metabolism, and xenobiotic metabolism, in the high-risk group decreased significantly (Figures 10A,B). Through the GO functional annotation of DEGs between different risk groups, we found that the genes upregulated in the high-risk group were mainly involved in nuclear division, mitotic nuclear division, chromosome segregation, and microtubule cytoskeleton organization during mitosis (Figures 10C,D), which also indicated that the molecular signature of the high-risk group was closely related to the cell cycle. In addition, the immune response of the high-risk group was lower than that of the low-risk group, as revealed by GSEA (Figure 10E). Interestingly, we also found that the activity of metabolism-related pathways was higher in the subtype with a good prognosis *via* pairwise comparison among the three subtypes (Figures 11A–F).

### External Validation of the Prognostic Signature and Clinical Correlation Analysis

**FIGURE 12:**
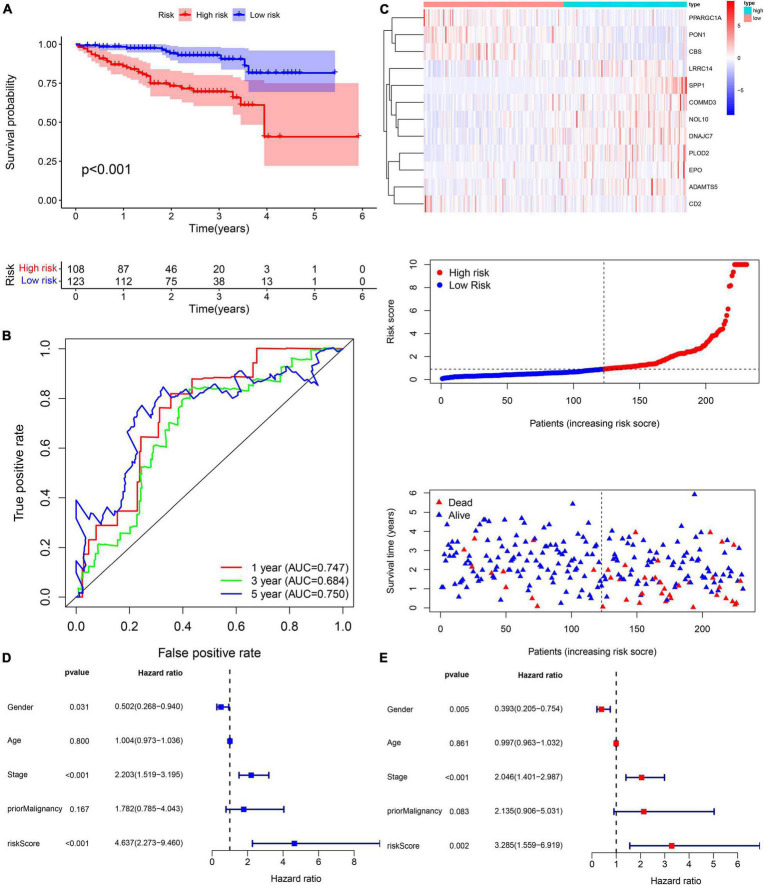
External validation of the risk score in the ICGC cohort. **(A,B)** The Kaplan–Meier survival analysis and the time-dependent ROC analysis for the risk score in predicting the OS of patients in the ICGC cohort. **(C)** The heatmap, risk score distribution, and survival status of patients in the ICGC cohort. **(D)** The forest plot of univariate Cox analysis. **(E)** The forest plot of multivariate Cox analysis.

The risk score of patients in the ICGC (*n* = 231) was calculated with the formula derived from the training cohort, and the patients were divided into high- and low-risk groups with the cutoff (0.9368) consistent with the training cohort. The high-risk patients’ OS was significantly reduced (Figure 12A). The AUC values of OS at 1, 3, and 5 years predicted by the risk score were 0.747, 0.684, and 0.750, respectively (Figure 12B). As the risk score increased, the number of deaths increased (Figure 12C). The risk score was an independent prognostic indicator for HCC as demonstrated by univariate and multivariate Cox regression analysis (Figures 12D,E). The chi-square test showed that the prognostic signature exhibited a close relationship with histology grade, vascular tumor cell type, AFP, new tumor event after initial treatment, main tumor size, cirrhosis, TNM stage, BCLC stage, and CLIP score (*p* < 0.05, Tables 1–3).

**TABLE 1:** The chi-square test of the relation between different risk groups and clinical features in TCGA cohort.

	**High risk**	**Low risk**	**Chi**	***P*-value**
**Age**				
≤65	72 (72%)	90 (66.18%)	0.6576	0.4174
>65	28 (28%)	46 (33.82%)		
**Gender**				
Female	34 (34%)	44 (32.35%)	0.0158	0.8999
Male	66 (66%)	92 (67.65%)		
**Histology grade**				
G1	5 (5%)	21 (15.44%)	24.0693	0
G2	35 (35%)	73 (53.68%)		
G3	51 (51%)	40 (29.41%)		
G4	9 (9%)	2 (1.47%)		
**TNM_stage**				
I	45 (45%)	89 (65.44%)	11.5671	0.0031
II	28 (28%)	30 (22.06%)		
III–IV	27 (27%)	17 (12.5%)		
AFP				
=300 ng/ml	66 (66%)	115 (84.56%)	10.0907	0.0015
>300 ng/ml	34 (34%)	21 (15.44%)		
**Vascular_tumor_cell_type**				
Macro	6 (6%)	4 (2.94%)	11.5266	0.0031
Micro	41 (41%)	31 (22.79%)		
None	53 (53%)	101 (74.26%)		
**New_tumor_event_after_initial_treatment**				
No	45 (45%)	82 (60.29%)	4.8255	0.028
Yes	55 (55%)	54 (39.71%)		

**TABLE 2:** The chi-square test of the relation between different risk groups and clinical features in GSE14520 cohort.

	**High risk**	**Low risk**	**Chi**	***P*-value**
**Gender**				
Female	12 (10.91%)	16 (14.95%)	0.4705	0.4927
Male	98 (89.09%)	91 (85.05%)		
Age				
≤65	104 (94.55%)	94 (87.85%)	2.2628	0.1325
>65	6 (5.45%)	13 (12.15%)		
ALT				
≤50 U/L	60 (54.55%)	67 (62.62%)	1.1422	0.2852
>50 U/L	50 (45.45%)	40 (37.38%)		
**Main tumor SIZE**				
≤5 cm	63 (57.27%)	77 (71.96%)	4.4912	0.0341
>5 cm	47 (42.73%)	30 (28.04%)		
Multitumor				
Multiple	28 (25.45%)	19 (17.76%)	1.4676	0.2257
Solitary	82 (74.55%)	88 (82.24%)		
**Cirrhosis**				
None	2 (1.82%)	15 (14.02%)	9.5559	0.002
Yes	108 (98.18%)	92 (85.98%)		
**AFP**				
≤300 ng/ml	56 (50.91%)	64 (59.81%)	1.3981	0.237
>300 ng/ml	54 (49.09%)	43 (40.19%)		
**Stage_TNM**				
I-II	74 (67.27%)	94 (87.85%)	11.9872	5.00E-04
III-IV	36 (32.73%)	13 (12.15%)		
**Stage_BCLC**				
0-A	74 (67.27%)	91 (85.05%)	8.454	0.0036
B-C	36 (32.73%)	16 (14.95%)		
**CLIP_Score**				
<2	78 (70.91%)	91 (85.05%)	5.4991	0.019
≥2	32 (29.09%)	16 (14.95%)		

**TABLE 3:** The chi-square test of the relation between different risk groups and clinical features in ICGC cohort.

	**High risk**	**Low risk**	**Chi**	***P*-value**
**Gender**				
Female	30 (27.78%)	31 (25.2%)	0.086	0.7693
Male	78 (72.22%)	92 (74.8%)		
**Age**				
≤65	40 (37.04%)	49 (39.84%)	0.0905	0.7635
>65	68 (62.96%)	74 (60.16%)		
**Stage TNM**				
I-II	53 (49.07%)	88 (71.54%)	11.2832	8.00E-04
III-IV	55 (50.93%)	35 (28.46%)		
**Prior malignancy**				
No	92 (85.19%)	109 (88.62%)	0.3343	0.5631
Yes	16 (14.81%)	14 (11.38%)		

### Therapeutic Response Prediction

We compared the sensitivity of several commonly used antitumor drugs between the high-risk group and the low-risk group, and the sensitivity of the low-risk group to sorafenib, rapamycin, and gefitinib was higher than that of the high-risk group (Supplementary Material 4). However, there was no significant difference in the response to immunotherapy between the two groups (Supplementary Material 4).

## Discussion

Hepatocellular carcinoma (HCC) has a high recurrence rate and mortality rate and lacks effective treatment, which seriously threatens human health (Yang et al., 2019). The occurrence and development of HCC is a very complex biological process. The improvement of clinical treatment practice depends on the in-depth exploration and interpretation of the biological essence of HCC. The traditional treatment of HCC is mainly surgery supplemented by postoperative radiotherapy, chemotherapy, and traditional Chinese medicine (Marks and Yee, 2016). In recent years, with the rapid development of science and the wide application of systems biology technologies such as genomics and proteomics, disease-related gene variation and signal pathway changes have been revealed through a high-throughput big data system, which provides new ideas and strategies for accurate classification, individualized treatment, efficacy monitoring, and prognosis judgment of diseases. Therefore, it has become a focus of clinical research to intervene in the progression of HCC at the molecular level, but drug resistance and immune escape are the main problems faced by targeted therapy and immunotherapy (Thomas et al., 2008). Controlling HCC by inducing cancer cell death is a common feature of treatment methods, and recent studies have shown that two newly discovered PCD types, ferroptosis and pyroptosis, have great significance in the treatment of HCC (Hage et al., 2019; Lippmann et al., 2020).

This study was carried out from the perspective of prognosis. First, we analyzed the prognostic value of regulatory factors related to ferroptosis and pyroptosis in HCC. A total of 99 genes were associated with the OS of HCC as shown by univariate Cox regression analysis (*p* < 0.05) and were considered to be PRGs. Based on the expression level of PRGs, the training cohort, which contained 609 HCC patients, was clustered into three subtypes. Through the pairwise comparison of the three subtypes, we found that there were significant differences in prognosis among C1, C2, and C3. To explore the molecular differences among different subtypes, we further identified the DEGs of the different subtypes. A total of 2,930 overlapping DEGs were speculated to have important prognostic significance. After screening by univariate Cox regression analysis and reducing the scope by LASSO regression analysis, 12 genes constituted the risk score derived from their multivariate Cox regression coefficients. The median risk score of the training cohort of 0.9368 served as the unified cutoff to divide patients into a high-risk group and a low-risk group. The Kaplan–Meier survival analysis suggested that the risk score could effectively distinguish patients with a favorable prognosis from patients with an adverse prognosis, and the time-dependent ROC curves showed that the risk score had good accuracy in predicting the OS, DSS, DFS, PFS, and RFS of HCC. Importantly, internal validation (TCGA, *n* = 370; GSE14520, *n* = 239) and external validation (ICGC, *n* = 231) confirmed that the 12-gene risk score was a stable and universally applicable prognostic assessment instrument for HCC. Interestingly, subgroup (*n* = 26) analysis showed that regardless of the clinical characteristics of HCC patients, the prognoses of the high-risk group and low-risk group were all significantly different. When they belonged to the low-risk group, their prognosis was relatively good. In contrast, when they belonged to the high-risk group, the prognosis was relatively poor, a difference that was statistically significant.

To investigate the prognostic mechanism of the proposed signature and reveal the causes leading to prognosis differences among the different risk groups, we compared different risk groups in terms of the tumor immune microenvironment (TIME), gene mutation, TMB, cancer stem cell (CSC) characteristics (mRNAsi), molecular signature, and clinical features. In terms of the TIME, several immune cells with different infiltration degrees between the high-risk group and the low-risk group, such as activated B cells, activated CD8 T cells, DCs, and type I T helper cells, had a significant impact on the OS of HCC. CD8 T cells are the most important effector cells against tumors, and activated CD8 T cells recognize tumor-related antigens on major histocompatibility complex I (MHC I) through their expressed T cell receptors to kill tumor cells (Gajewski et al., 2013). The high infiltration of CD8 T cells in the TIME is related to a favorable prognosis of breast cancer, lung cancer, melanoma, and colorectal cancer (Fridman et al., 2012; Farhood et al., 2018). This study showed that high infiltration of activated CD8 T cells was related to a good prognosis of HCC.

B cells are the main effector cells of humoral immunity, and they can promote the T-cell response by secreting immunoglobulin and directly killing tumor cells to inhibit the progression of tumors (Tokunaga et al., 2018). This study found that high infiltration of B cells in the TIME was related to a good prognosis of HCC. Type I T helper cells (Th1 s) are the most important helper cell type in tumor immunity. Th1 s can directly kill tumor cells by releasing cytokines that activate death receptors on the surface of tumor cells, enhance the CD8 T cell response, and contribute to the recruitment of natural killer cells and type I macrophages to the tumor (Knutson and Disis, 2005; LaCasse et al., 2011; Kim and Cantor, 2014). This study found that high infiltration of B cells in the TIME was related to a good prognosis of HCC.

DCs are the most powerful professional antigen-presenting cells (APCs) in the immune system. DCs play a key role in the initiation and regulation of the immune response. According to their mature state and function, DCs can be divided into immature DCs, regulatory or tolerant DCs, and mature DCs. Mature DCs can induce the body to produce a specific immune response and play an anti-infection and antitumor role. In contrast, immature DCs can further produce immune tolerance by inducing the body to produce regulatory T cells, anergic T cells, or tolerant T cells (Dhodapkar et al., 2001). This study found that high infiltration of immature DCs in the TIME was related to a poor prognosis of HCC.

The loss of human leukocyte antigen (HLA) gene function will lead to the loss of HLA-I surface expression on tumor cells, thus blocking the antigen-presenting pathway and mediating immune escape (No authors listed, 2018). This study found that the expression of HLA was significantly reduced in the high-risk group with a poor prognosis.

The TP53 gene encodes the tumor protein p53, which is a tumor suppressor that prevents cell division and proliferation (Donehower et al., 2019). This study found that TP53 mutation is related to a poor prognosis of HCC, and it has a high mutation rate in the high-risk group. The TMB is considered one of the biomarkers to predict the efficacy of immune checkpoint inhibitors (Choucair et al., 2020). This study found that high-risk patients with a poor prognosis had a higher TMB. Considering that TP53 mutations could cause increased genomic instability (Moore et al., 2005), the higher TMB in the high-risk group may be associated with the higher TP53 mutation rate in this group.

CSCs have self-renewal ability and can produce heterogeneous cancer cells, which play an important role in cancer survival, proliferation, metastasis, and recurrence (Gupta et al., 2009). By comparing mRNAsi, we found that the stem cell characteristics of cancer cells in the high-risk group were stronger, indicating that the ability of tumor cells to proliferate and induce angiogenesis in the low-risk group may be lower than that in the high-risk group. The positive correlation between the risk score and TMB and mRNAsi demonstrated that a high risk score resulted in enhanced tumor stem cell characteristics and significantly increased the TMB of HCC. We can predict the genomic instability and stem cell characteristics of HCC patients based on the risk score.

As for the molecular signature, with the help of GSVA and GSEA, we found that both the low-risk group with a good prognosis and the subtype with a good prognosis all belonged to the metabolic active type. An active metabolism was also revealed by scholars such as Gao to be one of the important signatures of a good prognosis of HCC (Gao et al., 2019), and the molecular mechanism of the high-risk group with a poor prognosis not only was related to low metabolic activity but also involved the immune response and cell cycle regulation. Clinically, vascular invasion, histological grade, TNM stage, BCLC stage, and other indicators are closely related to prognosis, and they are an important reference basis for judging tumor invasiveness and malignancy. Through the chi square test, we found that there were significant differences in the above clinicopathological features between the high-risk group and the low-risk group. Based on the above results, we summarized the characteristics of HCC patients with a poor prognosis: microenvironment immunosuppression, TP53 mutation, genomic instability (high TMB), stronger characteristics of CSCs (high mRNAsi), inactive metabolism, vascular invasion, advanced stage (TNM III-IV, BCLC B-C, and CLIP > 2), main tumor size > 5 cm, poor differentiation, and cirrhosis. These findings may provide guidance for a deeper understanding of the pathogenesis of HCC and the precise clinical diagnosis and treatment of HCC.

Some of these 12 genes have been previously proven to be closely related to the progression of cancer. For example, the prognostic value of ADAMTS5 is controversial in HCC. Zhu et al. (2021) found that high ADAMTS5 expression was significantly associated with a worse survival, which is consistent with our results, but Li et al. (2015) found that the expression of ADAMTS5 was inversely associated with the OS of HCC. Wang et al. (2018) found that cystathionine β-synthase (CBS) induces multidrug resistance and the metastasis of HCC. Kubo et al. (2013) found that DNAJC7 participates in the negative feedback regulation pathway of p53/MDM2, thereby enhancing the stability and activity of p53. Umbreen et al. (2019) suggested that COMMD3 participated in the progression of metastatic prostate cancer by regulating C-MYC transcription. Noda et al. (2012) showed that the overexpression of PLOD2 was closely related to a poor prognosis of HCC. Ding et al. (2020) found that serum PON1 could predict microvascular invasion of HCC. Kido and Lau (2019) found that the downregulation of PPARGC1A was correlated with a favorable prognosis for HCC patients. Wang et al. (2019) suggested that SPP1 may function as an enhancer of HCC growth targeted by miR-181c.

In conclusion, our study revealed for the first time a novel prognostic signature for HCC based on ferroptosis and pyroptosis molecular subtypes. The high-risk group and low-risk group were not only significantly different in their prognoses but also significantly different in the tumor immune microenvironment, tumor stem cell characteristics, TMB, TP53 mutation, molecular pathway, and clinicopathological characteristics. In future work, targeted treatment strategies and biomarkers will be further developed for each subgroup to maximize the curative effect and achieve individualized treatment of HCC based on molecular signatures.

## Conclusion

A robust signature identified and validated in this work based on ferroptosis and pyroptosis molecular subtypes can be used for survival prediction, immune microenvironment estimation, stem cell characteristics, and clinical feature assessment in HCC.

## Data Availability Statement

Publicly available datasets were analyzed in this study. This data can be found here: The datasets analyzed for this study were obtained from The Cancer Genome Atlas (TCGA, https://portal.gdc.cancer.gov/), International Cancer Genome Consortium database (ICGC, https://dcc.icgc.org/releases/current/Projects/LIRI-JP), Gene Expression Omnibus (GEO, https://www.ncbi.nlm.nih.gov/geo/), FerrDb (http://www.zhounan.org/ferrdb), and the molecular signatures database (MSigDB, http://www.gsea-msigdb.org/gsea).

## Author Contributions

JH and LW designed this study. GG and HL collected the data. JH analyzed the data in this study, interpreted the findings, and drafted the manuscript. LW and JC carried out data management and revised the manuscript. All authors reviewed the final version of the manuscript.

## Conflict of Interest

The authors declare that the research was conducted in the absence of any commercial or financial relationships that could be construed as a potential conflict of interest.

## Publisher’s Note

All claims expressed in this article are solely those of the authors and do not necessarily represent those of their affiliated organizations, or those of the publisher, the editors and the reviewers. Any product that may be evaluated in this article, or claim that may be made by its manufacturer, is not guaranteed or endorsed by the publisher.

## Footnotes

1
https://portal.gdc.cancer.gov/

2
https://icgc.org/

3
https://www.ncbi.nlm.nih.gov/geo/

4
http://www.gsea-msigdb.org/gsea

5
http://www.zhounan.org/ferrdb

## Supplementary Material

Supplementary Image 1 The work flow chart.

Supplementary Image 2 The correlation between the risk score and therapeutic response prediction.

## Abbreviations

HCC: hepatocellular carcinoma
TCGA: The Cancer Genome Atlas
ICGC: International Cancer Genome Consortium
GEO: Gene Expression Omnibus
PRGs: prognostic related genes
DEGs: differential expressed genes
GO: Gene ontology
KEGG: Kyoto Encyclopedia of Genes and Genomes
KM: Kaplan–Meier
LASSO: least absolute shrinkage and selection operator
ROC: receiver operating characteristic
AUC: area under curve
OS: overall survival
DSS: disease-specific survival
DFS: disease-free survival
PFS: progression-free survival
RFS: relapse-free survival
GSVA: Gene Set Variation Analysis
NES: normalized enrichment score
GSEA: Gene Set Enrichment Analysis
ssGSEA: single sample gene set enrichment analysis
IFN: interferon
TIME: tumor immune microenvironment
TMB: tumor mutation burden
CSCs: cancer stem cells
HLA: human leukocyte antigen.
