# Supplementary figures and images for: A Ferroptosis and Pyroptosis Molecular Subtype-Related Signature Applicable for Prognosis and Immune Microenvironment Estimation in Hepatocellular Carcinoma
Source: Front Cell Dev Biol. 2021 Nov 15;9:761839. doi: 10.3389/fcell.2021.761839 (PMC8634890; doi:10.3389/fcell.2021.761839)

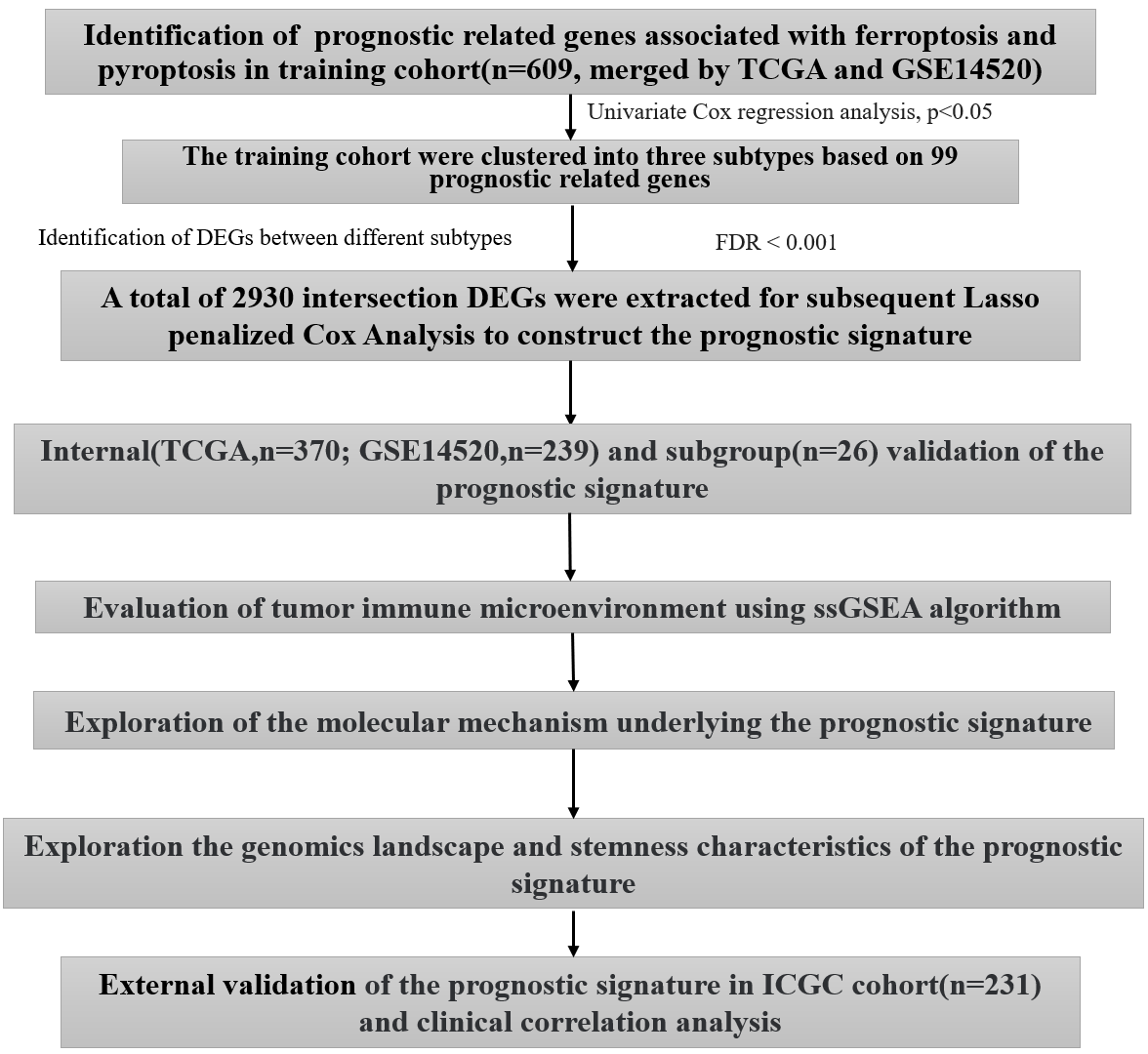

Supplement: Supplementary Image 1 — The work flow chart. [file Image_1.PNG]
